# Fluoro-forest: a random forest workflow for cell type annotation in high-dimensional immunofluorescence imaging with limited training data

**DOI:** 10.1093/bioadv/vbaf320

**Published:** 2025-12-24

**Authors:** Joshua Brand, Wei Zhang, Evie Carchman, Huy Q Dinh

**Affiliations:** McArdle Laboratory for Cancer Research, Department of Oncology, School of Medicine and Public Health, University of Wisconsin-Madison, Madison, WI 53705, United States; Department of Pathology and Laboratory Medicine, The University of Kansas Medical Center, Kansas City, KS 66103, United States; Department of Surgery, School of Medicine and Public Health, University of Wisconsin-Madison, Madison, WI 53705, United States; Department of Surgery, William S. Middleton Memorial Veterans Hospital, Madison, WI 53705, United States; University of Wisconsin Carbone Cancer Center, University of Wisconsin School of Medicine and Public Health, Madison, WI 53705, United States; McArdle Laboratory for Cancer Research, Department of Oncology, School of Medicine and Public Health, University of Wisconsin-Madison, Madison, WI 53705, United States; University of Wisconsin Carbone Cancer Center, University of Wisconsin School of Medicine and Public Health, Madison, WI 53705, United States; Department of Biostatistics and Medical Informatics, School of Medicine and Public Health, University of Wisconsin-Madison, Madison, WI 53705, United States

## Abstract

**Motivation:**

Cyclic immunofluorescence (IF) techniques enable deep phenotyping of cells and help quantify tissue organization at high resolution. Due to its high dimensionality, workflows typically rely on unsupervised clustering, followed by cell type annotation at a cluster level for cell type assignment. Most of these methods use marker expression averages that lack a statistical evaluation of cell type annotations, which can result in misclassification. Here, we propose a strategy through an end-to-end pipeline using a semi-supervised, random forest approach to predict cell type annotations.

**Results:**

Our method includes cluster-based sampling for training data, cell type prediction, and downstream visualization for interpretability of cell annotation that ultimately improves classification results. We show that our workflow can annotate cells more accurately compared to representative deep learning and probabilistic methods, with a training set <5% of the total number of cells tested. In addition, our pipeline outputs cell type probabilities and model performance metrics for users to decide if it could boost their existing clustering-based workflow results for complex IF data.

**Availability and implementation:**

Fluoro-forest is freely available on GitHub under an MIT license (https://github.com/Josh-Brand/Fluoro-forest).

## 1 Introduction

High-plex immunofluorescence (IF) imaging has significantly expanded our ability to visualize and characterize cell types and subsets, offering detailed views of tissue-level biology. While these technologies have existed for years (Schubert 2003, Lin *et al.* 2015, Goltsev *et al.* 2018), their accessibility through commercial platforms has significantly improved. Imaging-based antibody panels can now exceed 50 markers and are increasingly used, as seen with Codex (Lin *et al.* 2015), but open-source tools to accurately label cell types remain needed. Additionally, these datasets may span tens to hundreds of thousands of cells, making conventionally supervised or threshold-based approaches that use complex combinations of markers for annotation infeasible. Open-source software, such as ImageJ (Schindelin *et al.* 2012) and QuPath (Bankhead *et al.* 2017), are excellent tools with extended functionality through plugins for cell segmentation, feature calculation, and cell annotation. QuPath’s classification algorithms include random forests; however, it predicts single marker positivity and was not designed to generate multi-class models. While these tools exist to help guide annotation and image analyses, multi-class annotations have been favored using third party tools in Python or R.

Other machine learning approaches include a probabilistic Bayesian framework *Celesta* (Zhang *et al.* 2022) and deep learning approaches such as *MAPS* (Shaban *et al.* 2024) and *CellSighter* (Amitay *et al.* 2023) for cell type classification. Celesta uses a design matrix to guide cell type annotation and models the data using all provided cells, rather than requiring an independent training set. This approach assumes that some markers will not overlap but may struggle where fluorescence bleedover from areas with high cell density. Conversely, MAPS and CellSighter require large amounts of training data, potentially thousands to tens of thousands of cells, provided by other cell-type annotation methods. Unsupervised methods such as clustering, is commonly applied to other single-cell omics technologies, however its application to imaging data remains challenging (Hickey *et al.* 2021). Aggregate expression profiles of clusters are difficult to interpret due to background signal in imaging data, non-normal data distributions, and varying cluster resolutions that may mask rare cell types while over-clustering others. Evaluation of clustering-based and other cell-type annotation methods still requires a well-labeled training dataset generated with expert knowledge, which remains a consistent bottleneck. Our approach shows that small training datasets (200–300 cells) from a typical 2000–10 000 cell core biopsy can significantly improve cell type annotation results and may be a better approach than deep learning when training data is limited.

## 2 Methods

**Figure 1.**
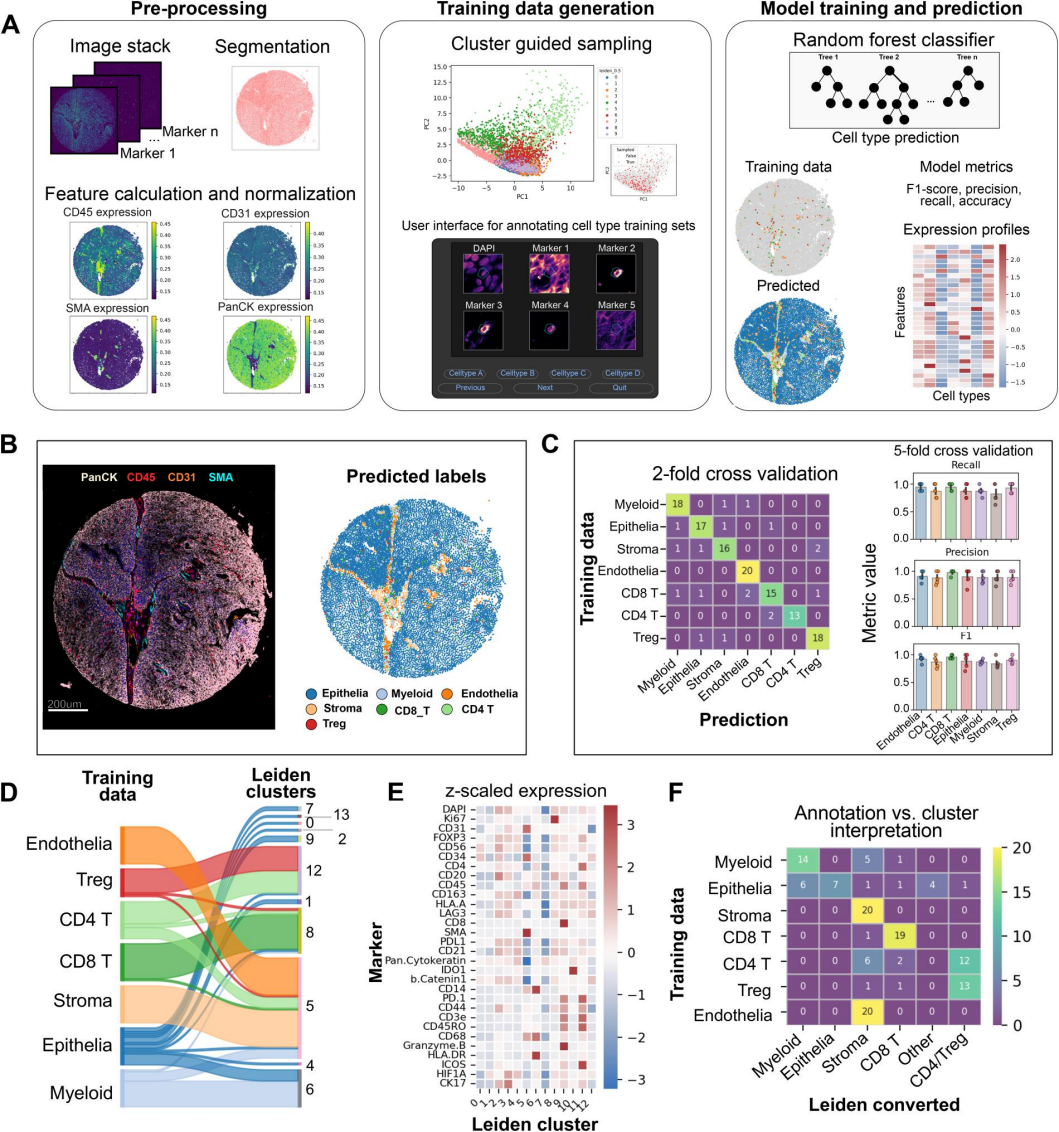
Fluoro-forest: A semi-supervised cell type annotation method using random forests for high-plex IF data. (A) Our workflow converts images to tabular data, guides cell annotation to train random forest models for predicting unseen data. (B) Lineage markers for epithelia (PanCK), endothelia (CD31), smooth muscle/stroma (SMA), and immune cells (CD45) supports predicted annotations. (C) Classes were balanced prior to training and tested for model accuracy in two- and five-fold cross validation, achieving 87% and 90.4% ± 2.7% accuracy, respectively (mean ± SD). (D) Sankey diagram of annotated cell types and their Leiden-based clustering. (E) *Z*-scored expression values for each Leiden cluster across all markers. (F) Expression guided annotation from clustering was unable to split CD4+ T cells from Tregulatory cells, and stroma from endothelia.

We developed Fluoro-forest, an end-to-end Python pipeline for cell type annotation and prediction using user-defined protein markers. Our pipeline begins with cell segmentation using existing methods, such as StarDist (Schmidt *et al.* 2018) or CellPose (Stringer *et al.* 2021) to identify cell boundaries (Fig. 1A, Fig. 1, available as supplementary data at *Bioinformatics Advances* online). The segmentation allows cells to be represented as tabular data, where pixel intensities are averaged in segmentation masks. These values are then log2-transformed, with the extreme values clipped and standardized before clustering (Methods, available as supplementary data at *Bioinformatics Advances* online). The images (one slice per marker), tabular expression data, and segmentations are inputs for a Python class for data visualization, cell sampling for training data, annotation, and cell type prediction. In our workflow, the user is prompted to select which markers (by default, all) to use in dimension reduction with principal components analysis (PCA) for visualization. We support unsupervised clustering methods (k-means, Louvain, Leiden, or Gaussian mixture models) to initialize the sampling procedure to ensure a diverse training dataset across the cell types represented in a tissue of interest. The sampled cells will be annotated by users through a multichannel viewing window and serve as training data for a random forest model to predict the remaining unannotated cells. We include optional settings such as imbalanced random forests or synthetic sampling via SMOTE (Chawla *et al.* 2011) to improve classification accuracy in cases where the majority cell type dominates the training data. Finally, we provide cross-validation performance metrics (precision, recall, and F1 scores) output per cell type to guide model tuning.

## 3 Results

**Figure 2.**
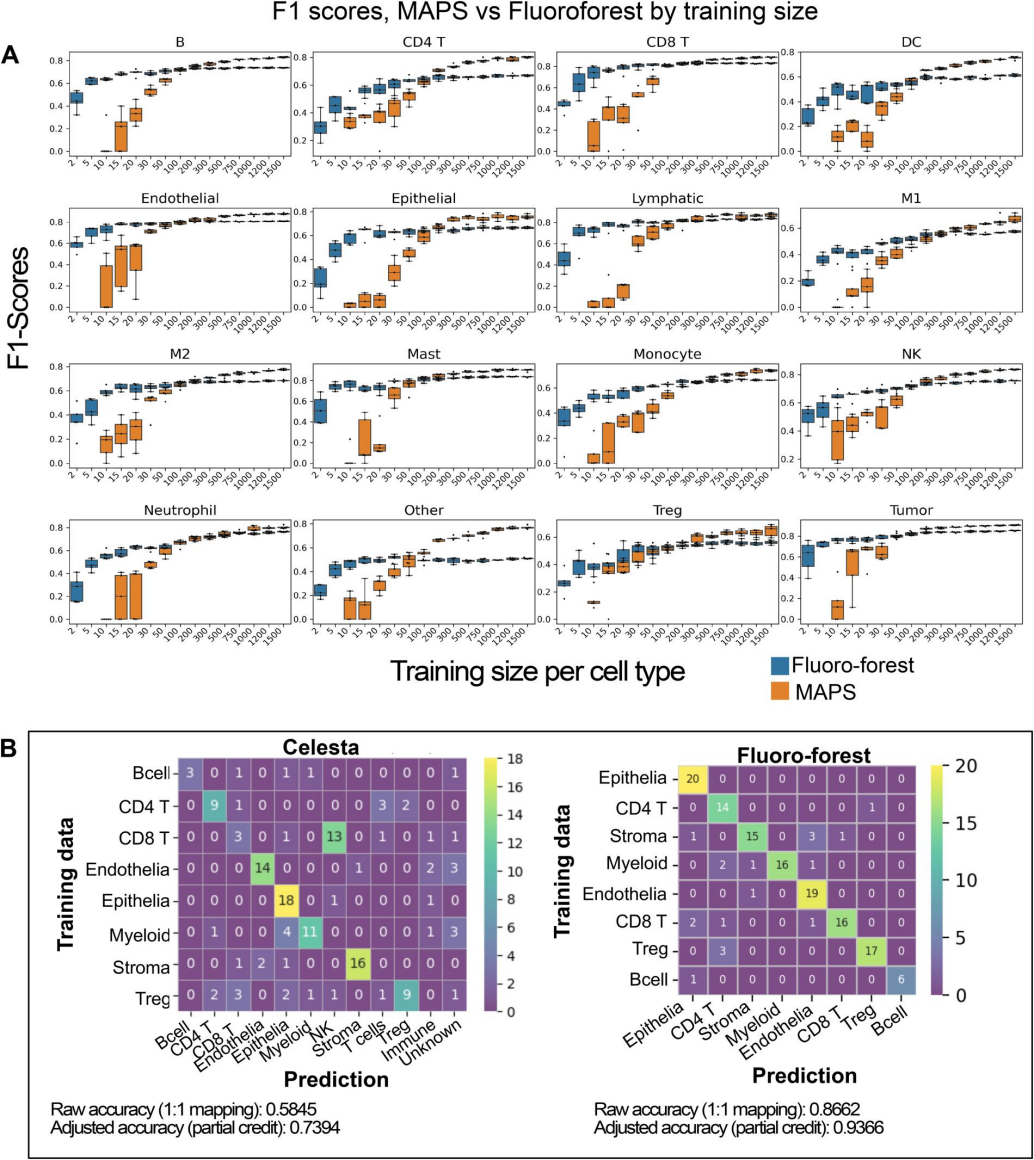
Fluoro-forest makes accurate predictions with significantly less training data relative to deep learning approaches. (A) Public Codex data shows the effect of training size on F1 scores using five-fold cross validation. Models were evaluated on a held-out dataset of >28 000 cells. (B) Contingency plot comparing Celesta’s predicted cell labels versus Fluoro-forest predicted labels from an in-house Codex dataset. Celesta’s hierarchical model generates new labels based on its uncertainty (e.g. Immune, Unknown, T cells).

To illustrate the workflow, we applied our pipeline to PhenoCycler/Codex data from two in-house anal dysplasia biopsies with an immune-focused protein panel (*N* = 30 markers) (Fig. 1B, Fig. 2A, available as supplementary data at *Bioinformatics Advances* online). We identified cell types in these datasets, including tumor/epithelial cells, stroma, endothelial cells, myeloid, CD4 and CD8 T cells, B cells, and Tregs based on their representative markers (Methods, available as supplementary data at *Bioinformatics Advances* online) with lineage marker expression supporting our final cell type predictions (Fig. 1B, Fig. 2A, available as supplementary data at *Bioinformatics Advances* online).

### 3.1 Fluoro-forest accurately predicts cell types from minimal training sets

Five-fold cross validation showed high accuracy (90.4% ± 2.7%, mean ± SD) from our included data (Fig. 1C) and demonstrates the model’s ability to identify true classes while minimizing false positives and false negatives, achieving F1 scores of 0.75 or higher. We compared our pipeline to annotations derived from a Leiden-based clustering workflow and found consistent cell-type mixing within our clusters. For instance, Leiden cluster 5 includes a mixture of endothelial, epithelial, CD4 T, and stroma cells (Fig. 1D), which poses a challenge for annotation based on average marker expression (Fig. 1E and F). We demonstrate similar challenges with a second independent biopsy (Fig. 2B–D, available as supplementary data at *Bioinformatics Advances* online). Specifically, we used all features to over-cluster the data, which is recommended (Hickey *et al.* 2021) to recover less abundant cell types, such as Tregs and myeloid cells. Leiden clustering was able to identify distinct subsets within epithelial cells but failed to differentiate FOXP3+ T regulatory cells from other CD4+ T cells and CD31+ endothelial cells from SMA+ stromal cells (Fig. 1F, Fig. 2D, available as supplementary data at *Bioinformatics Advances* online), suggesting the need for supervised machine learning approaches.

### 3.2 Fluoro-forest performs better in comparison with existing methods for limited training data

We sought to evaluate the predictive performance of Fluro-forest compared with the latest deep learning method, *MAPS*, and a probabilistic method, *Celesta*. MAPS has been shown to outperform other existing deep learning methods for cell type classification (Shaban *et al.* 2024), but its application depends on the feasibility of generating large volumes of training data. First, we compared MAPS to Fluro-forest models using five-fold cross-validation with increasing training data sizes using the MAPS authors’ expert-annotated Codex data derived from lymphoma samples. The randomized training data were made consistent between both model types, and the final models were used to predict cell type pe labels using a withheld validation set of >28k cells.

We show that Fluro-forest can generate comparable predictions with much less data compared to MAPS, which often required >100 training examples per class before seeing improved prediction performance (Fig. 2A). Specifically, Fluro-forest outperforms MAPS for all cell types with <100 cells per cell type in the training data, except Tregs, where the performance is more comparable. Naturally, for the deep learning method, MAPS performs better when the training data exceeds 100 training samples per cell type. We note that annotating beyond 100 cells per is time-consuming and may not be possible in smaller datasets where rare cell types may not exceed 50 cells. We also show that a random forest model with as few as 30 cells per class, a modest training set size for an individual to generate manually, demonstrates more robust precision-recall curves compared to MAPS (Fig. 3A and B, available as supplementary data at *Bioinformatics Advances* online) with significantly faster training times (single-threaded tests on 32 GB RAM, i5 2 GHz processor, Fig. 3C, available as supplementary data at *Bioinformatics Advances* online).

Next, we tested the minimum samples per cell type needed for MAPS to outperform Fluoro-forest (Fig. 3D, available as supplementary data at *Bioinformatics Advances* online), for most cell types was >100 cells. We highlight this to illustrate the typical use case, in which a user may not have extensive pre-labeled data, and a well-performing model can be achieved with 30 cells per class for most cell types. We next tested the performance of Celesta using our in-house dataset (Fig. 2B), with our existing annotations (Fig. 2A and B, available as supplementary data at *Bioinformatics Advances* online). The contingency plots show the hierarchical method of annotation in Celesta, where, in cases of uncertainty, the model generates broader parent classes such as immune or T cells. To benchmark Celesta against Fluoro-forest, we applied a partial credit scheme if the prediction was a parent of the correct predicted class (Methods, available as supplementary data at *Bioinformatics Advances* online). Given its ability to offer reasonable probabilistic assignment of cells, and not just clusters, we see this could be a viable preprocessing step to rapidly generate training labels and correct errors before training other models, such as random forests, to boost performance.

## 4 Conclusion

Semi-supervised methods offer several advantages over clustering for cell type annotation in IF data. We see improved accuracy and generate interpretable probability outputs from trained models, i.e. the confidence probability of cell type annotation for a cell, which are not found in most clustering approaches. Our study shows that sampling approximately 20–30 cells per class was sufficient for high-accuracy predictions and performed better with modest training data sizes compared to deep-learning methods. However, when the training data is large enough, deep learning methods are recommended for these tasks. Despite these improvements, limitations exist. Annotation of rare cell types can still be challenging with our sampling strategy, and future implementations using active learning may improve this. Additionally, training data and, therefore, validation during data splits are represented by cells we can confidently annotate, but we acknowledge that there are areas in tissue where high cell density prevents confident annotation. Therefore, our model’s results depend on the quality of the training data, as with most machine learning methods. We expect that the model’s probability thresholds can be applied for a conservative estimate of cell types where this is available.

Ultimately, we expect these models may not generalize well across samples, showing significant accuracy loss (from >90% to <70%) (Fig. 4A–C, available as supplementary data at *Bioinformatics Advances* online) when trained on one core and predicting on the other, which was not remedied by different normalization procedures. When training data are generated from both cores, there is a slight loss in performance, with ∼88% accuracy in our cross-validation. We expect batch variation and donor heterogeneity to be challenges that could be overcome with additional feature engineering. In addition, other machine learning and preprocessing and normalization modules could be tested in our pipeline in future work to determine which are best suited to each step. Overall, our method offers a valuable, streamlined user interface appropriate for high-resolution and high-plex cell annotation.

## Supplementary Material

vbaf320_Supplementary_Data

## Data Availability

The data underlying this article are available on Dryad (Fluoro-forest CODEX data for random forest-based cell type annotation) and can be accessed under the following doi: 10.5061/dryad.hqbzkh1v1.
