# Supplementary material for: Fluoro-forest: a random forest workflow for cell type annotation in high-dimensional immunofluorescence imaging with limited training data
Source: Bioinform Adv. 2025 Dec 24;6(1):vbaf320. doi: 10.1093/bioadv/vbaf320 (PMC12782655; doi:10.1093/bioadv/vbaf320)
Supplement: vbaf320_Supplementary_Data [file vbaf320_supplementary_data.pdf]

Supplemental Methods

Data and code access

PhenoCycler / Codex data access for the 2 cores used in this manuscript is provided through Dryad (DOI: 10.5061/dryad.hqbzkh1v1). This submission includes images, segmentation results after applying StarDist[1], and QC'd expression values that are used for random forest training and prediction.

Fluoro-forest is freely available on GitHub (https://github.com/Josh-Brand/Fluoro-forest).

Our pipeline is provided through Jupyter Notebooks found at https://github.com/Josh-Brand/Fluoro-forest, and includes .yml files for environment setup, relying on standardized packages – scipy, numpy, matplotlib, sklearn, seaborn for data processing, visualization, and analysis. Our GitHub readme contains details on where to store the image files (N-7.ome.tif, C-12.ome.tif) and expression '_expression.tsv' files within the directory to reproduce our examples.

To reproduce the results, a .yml file is provided. After downloading the source code from GitHub, the images and expression summaries from dryad create a new virtual environment:

conda env create -f cell_annotation.yml

Enter into the project directory and run jupyter notebook / jupyter lab to access your notebooks. Proceed with running core-c7.ipynb, which walks through each step of data loading, clustering, sampling, annotation, and predictions.

Case study data

Two 2x2 cores for methodology evaluation were selected from a tumor microarray (TMA) with spatial phenotyping analysis using the Akoya Phenocycler Fusion (formerly known as Codex) using a custom panel (below). This approach uses tissue based cyclic immunofluorescence for highly multiplexed immunofluorescence imagining on FFPE specimens from glass slides. Final stitched images for the cores used here are available via Dryad in as .ome.tif files which were used for cell annotation.

30 maker panel: each image slice corresponding to the marker in the order shown

DAPI, Ki67, CD31, FOXP3 ,CD56	**1 - 5,**
CD34, CD4, CD20, CD45, CD163	**6 - 10,**
HLA-A, LAG3, CD8, SMA, PDL1	**11 - 15,**
CD21, PanCK, IDO1, bCat1, CD14	**16 - 20,**
PD-1, CD44, CD3e, CD45RO, CD68	**21 - 25,**
GZMB, HLA-DR, ICOS, HIF1A, CK17	**26 - 30**

Images and figures

Images presented in this paper were generated in QuPath[3] (v0.5.0) and pseudo-colored to show marker localization for DAPI, PanCK, SMA, CD31, and CD45.

Cell segmentation

Our workflow assumes cell segmentation has been performed on a multiplex IF image, where each image slice is an independent marker (**Methods Figure 1)**. The segmentation boundaries are converted to polygons and visualized during the cell annotation procedures.

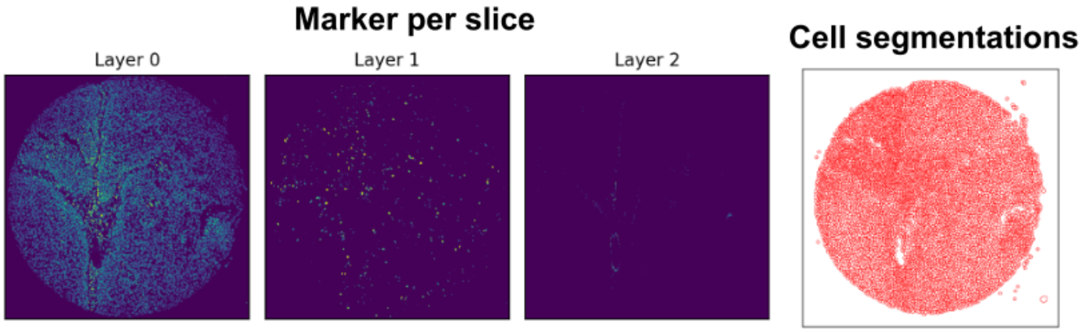

Methods Figure 1. Expected image layout requires each marker to be unique for each image to calculate expression summaries for each marker independently after segmentation.

In our examples, we applied a StarDist[1], a deep learning-based segmentation method using their existing nuclear segmentation model (dsb2018_heavy_agument.pb) provided via the QuPath plugin (https://github.com/qupath/qupath-extension-stardist). Channel values were normalized to 2-98 percentiles and a pixel size of 0.5 and cell expansion of 5 was applied. Thresholds for detection using StarDist were most sensitive to changing segmentation results and therefore tested at various levels and confirmed visually before exporting the data.

The segmentation results were saved out as a .geojson file using QuPath. The .json structures the cell ids and coordinates which are parsed using our utility functions before being read into into a python class for cell annotation, model training, and prediction **(Methods Figure 2**). The cell segmentations were used to summarize the expression of all markers creating features for cytoplasmic, membrane, nuclear, and cell regions as provided through QuPath's 'Measurement Export' tool. Our downstream analysis only considers features calculated over the entire segmentation region (suffix with '_Cell_Mean') other than DAPI, which was excluded in cell type prediction.

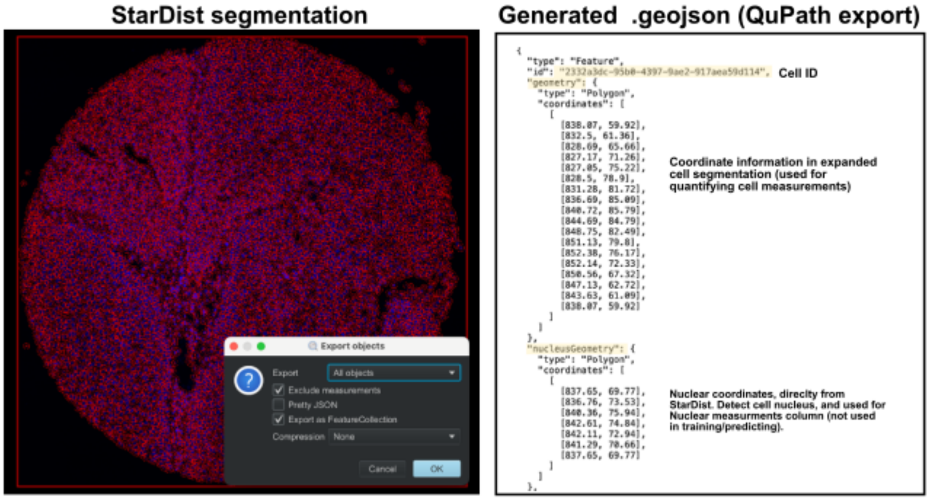

Methods Figure 2. QuPath segmentation after running StarDist generates a .geomjson file, which is parsed for individual cells and used to link expression data back to cell id and its location in tissue.

Note:
Our code base provides a small example of how to run segmentation exclusively in python to help new users, through an alternative cell segmentation workflow via CellPose[4], but requires a separate python environment with .yaml provided.

conda env create -f cellpose.yaml

After creating this environment activate it and start with the 'segmentation_example.ipynb' for a step-by-step example of cell segmentation. From this environment one of the cores is read in and cropped to generate a small data example. CellPose runs its internal nuclear model for segmentation and expression results are averaged over the entire cell, which are visualized to confirm its matching expression location in tissue.

Computational requirements

Training random forests is highly scalable even among tens of thousands of cells, allowing for hyperparameter evaluation across hundreds of folds. We performed all analyses stated here using a 2020 MacBook Pro i5 processor and 32Gb RAM. Each method run uses single-threads and was sufficient to run both our method as well as Celesta, and MAPS efficiently. While MAPS did take longer to run at higher sample sizes, the limitation was not restrictive. The MAPS dataset we applied our method to included 50 markers, which is on the higher level of what is possible to generate in multiplex immunofluorescence data. Pre-processing steps such as data transformation, scaling, principal components analysis, and clustering were not bottlenecks even as data scaled to tens of thousands of cells.

Data transformation

Segmentation boundaries were used to define cell ids and each pixel for each figure were summed and averaged across the segmentation mask using QuPath. The features calculated after segmentation were exported, along with cell ID's, and the centroids of cells (x, y spatial location). Each core was processed independently for QC analysis. Extreme values beyond the 1st and 99th percentiles of mean fluorescence intensity were clamped per feature (to the 1st, 99th

percentiles respectively), similar to existing workflows[5] to prevent the influence of outliers on mean expression. Next, we applied a log2 transform on these values with a pseudocount of 0.01 to reduce skewing and promote a more normal-like distribution. Log normalized intensity was used directly for random forest training and prediction but are standard scaled (value-mean(values)) / std(values) as is common for clustering and principal components analysis.

QC was performed to remove cells with poor quality segmentations, detected by low DAPI stain and under-stained cells. The QC'd data was read into a python notebook along with the image file (.tif), segmentation file (.geomjson from StarDist) for interactive cell annotation.

Clustering and cell sampling

Clustering via kmeans, Leiden, Louvain, and gaussian mixture models, are options provided to the user. As the workflow uses clustering to promote sampling diversity, the major consequence of unbalanced clustering is the amount of time one will spend annotating. We suggest applying a resolution parameter (k , resolution, number mixture components) to achieve more clusters than the number of major cell types that exist in the dataset to achieve diversity. By default, all non-nuclear stains are used generate clusters but can be subset to include some features if some stains are less informative. In our examples we applied Leiden clustering at a resolution of 0.8 from our standard scaled data and all cell stains to improve sampling. In cases where immune cells are expected to be more diverse (immune cells) we perform broad clustering using lineage markers and subscluster at higher resolutions and demonstrated in our jupyter notebooks.

Sampling procedures can be performed manually – through thresholding of markers to enrich for cell types. The workflow applied here was first performing a lineage-based clustering from SoftMax expression of lineage markers (PanCK, CD31, SMA, and CD45) and performing kmeans, with k = 4. The softmax procedure takes our log-transformed data, applies exponentiation to each element, and then normalizes these dividing each by the sum of all exponentiated values, and converting the output into something similar to a probability distribution. Later we applied Leiden clustering within each lineage level cluster to control cell type resolutions for harder to sample cell types or where more diversity is expected to exist. We oversample within each cluster to generate a diverse training queue and annotate high quality cells with clear marker expression and minimal cell overlap. For our data we selected markers to view and annotated according to **Methods Table 1**.

Methods Table 1. Marker combinations for cell type annotation.

	Epithelia	Endothelia	Stroma	CD4 T	CD8 T	Treg	NK	B cell	Myeloid
DAPI	+	+	+	+	+	+	+	+	+
PanCK	+	-	-	-	-	-	-	-	-
CK17	+/-	-	-	-	-	-	-	-	-
CD31	-	+	-	-	-	-	-	-	-
SMA	-	-	+	-	-	-	-	-	-
CD45	-	-	-	+	+	+	-	+	+
CD3e	-	-	-	+	+	+	+/-	-	-
CD4	-	-	-	+	-	+	-	-	+/-
CD8	-	-	-	-	+	-	+/-	-	-
FOXP3	-	-	-	-	-	+	-	-	-
CD14	-	-	-	-	-	-	-	-	+/-
CD68	-	-	-	-	-	-	-	-	+/-
CD163	-	-	-	-	-	-	-	-	+/-
HLA-DR	-	-	-	-	-	-	-	-	+/-
CD56	-	-	-	-	-	-	+	+/-	-
CD20	-	-	-	-	-	-	-	+	-

During annotation the samples selected based on clustering or any other means are provided to the user one at a time. A multi-channel viewing window is displayed with user-defined markers (**Methods Figure 3**) and annotation buttons allowing to annotate the cell type, skip to another cell, or exit the application. Upon quitting or finalizing annotations, the cell ids and their annotations are saved to the python class for later use in training and prediction.

Cluster annotation from average expression

Expression information was visualized using heatmaps by averaging the log-normalized expression of all values within a given annotation or cluster. These values were z-scored across annotations to demonstrate intensity relative to other groups for each marker. We used knowledge of combination and exclusive markers to attempt accurate classification with expression summaries, as is done in common workflows.

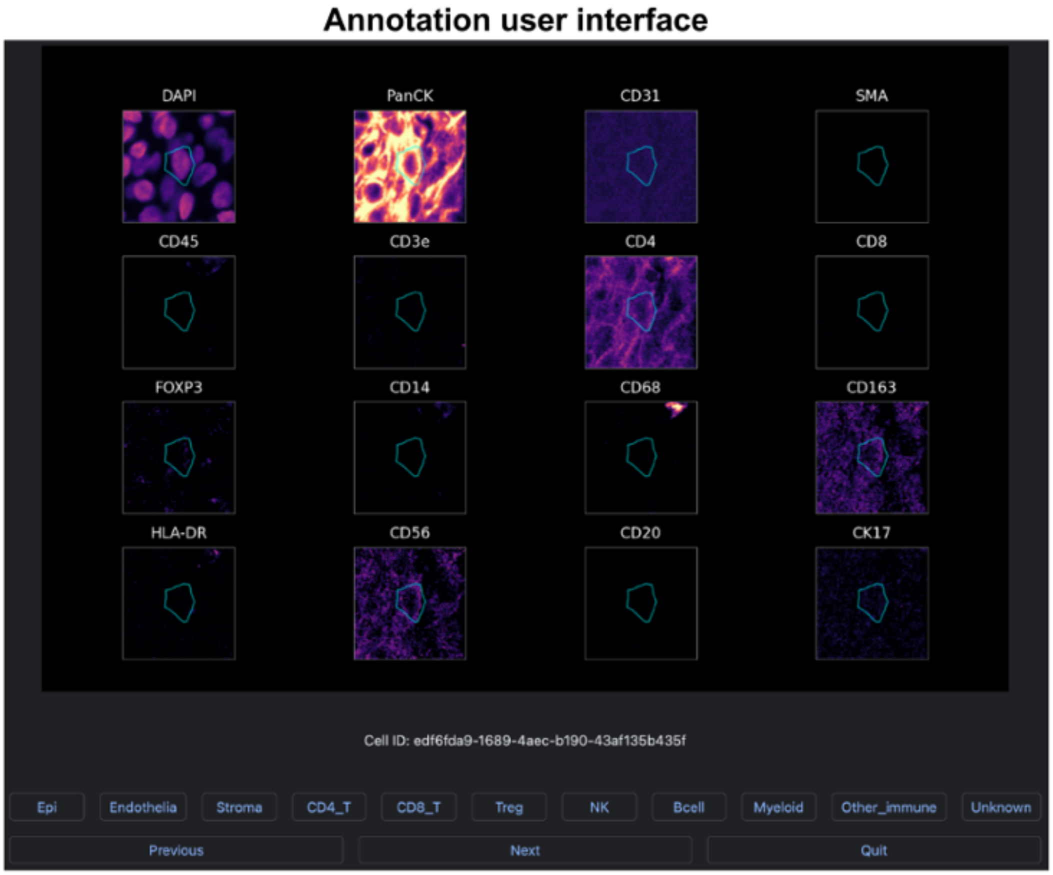

Methods Figure 3. Segmentations processed from .geomjson files are used to find individual cells in the image and are centered within each window. The cyan polygon represents the cell type to be annotated based visualized across channel markers in the dataset.

Random forest hyperparameters and tuning

For our in-house performance evaluation, random forests were trained with the following parameters: n_estimators=200, max_depth = None, min_samples_split = 2, min_samples_leaf=1, and max_features = ‘sqrt’. As these provided stable results across cross-

validation, further hyperparameter tuning was not performed on our own data as to not bias the results by optimizing for parameters in data that would likely be resampled in our training validation splits.

In larger datasets, such as that provided by MAPS, allowed for parameter tuning, which is foundin our rf_paramter_tuning notebook. Here we applied nested cross validation approaches in a grid search at increasing tree sizes, depth sizes, number of splits to use. Macro-F1 scores for models were used applied to see where performance could be gained and suggested larger models would perform better. Overall larger models resulted in small performance gains and models with 200-250 trees still performed well with a potential reduced risk of overfitting. Our final model applied in the MAPS dataset was as follows:

```
# random state for reproducibility, updated hyperparameters
    rf = RandomForestClassifier(n_estimators = 250, max_features = 'sqrt',
                                max_depth = 10, min_samples_leaf = 2,
                                min_samples_split = 5, random_state = 5 + i)
```

Random forest training, prediction, and performance evaluation

Cells were annotated across each core individually, guided by the clustering steps prior. Annotations were randomly down sampled to achieve a balanced representation of immune classes (B cells, myeloid, CD4 T, CD8 T, and Tregs) relative to epithelia, which were dominant in both cores. We down sampled to achieve approximately 40 cells per class, which was split into different cross validation (2 and 5-fold cross validation tests) schemes. 2-fold cross validation was used to visualize where misclassifications were occurring, while 5-fold cross validation was used to estimate model metrics such as precision: TP/(TP+FP), recall: TP/(TP+FN), and f1 scores: (2*precision*recall)/(precision + recall) for each. (TP =True positive, FP = False positive, FN = False negative).

The final predicted annotations were derived from a full model, which used the ~40 cells per class to predict the remaining unseen cells in our data. Last, we checked the performance of models trained on one core exclusively, then tested on the other or a composite model where cells from both cores were trained. The significant loss in accuracy when comparing the exclusive models suggest that data should be sampled across all cores where available for improved cell typing. Although the composite model performed with ~85% accuracy, the exclusive models support there are likely batch effects preventing high-quality, generalizable models without additional feature engineering.

Comparison to existing methods

We compared a random forest implementation to 2 existing methods, Celesta[5], which requires no training data labels and MAPS[6], which requires extensive training data.

Celesta, an R package, was installed and applied directly to our cores' log-normalized expression data. This required the expression matrix, x and y coordinates of cell centroids, and a design matrix which describes the specificity of cell type marker expression. As Celesta uses the entire dataset for inference and no training examples, we directly compared our annotation results from our 50/50 model split. A hierarchical scoring system was generated to compare the

accuracy of the models (celesta_predictions.ipynb). We assessed models both using standard F1, precision, and recall scores with exact matches, which over-penalized the results of Celesta. To compensate we applied a hierarchical scoring method, which attributed credit for correct parent-level predictions based on the design matrix hierarchy. Partial accuracy scored both exact matches and instances where the prediction belongs to the expected parent node.

We relied on the authors' generated data and annotations when running MAPS. To test a 'saturating' limit of samples needed for model accuracy, we employed a subsampling strategy to which matched training sets to compare random forest models to MAPS with 5-fold cross validation. Specifically, we sampled up to 1500 cells per class at increasing levels for a total of 16 classes and a constant withheld dataset containing more than 28,000 cells. We ran the existing parameters for MAPS that the authors' initially applied using an ADAM optimizer, a learning rate of 0.001, running for a minimum of 250 epochs, a maximum of 500 epochs, and patience of 100, which meant the model would hit an early stopping criteria if loss was not sufficiently reduced in the past 100 iterations.

MAPS failed to run with fewer than 10 cells, but the remaining iterations were used for per cell type F1 scores. Precision recall curves were generated (sklearn.metrics PrecisionRecallDisplay function) for models trained with 30 samples. This number was chosen because the improvements in random forests tend to taper off after 30 trainings examples and represents a reasonable number of cells for someone to annotate in their own dataset.

References

1. Schmidt, U., Weigert, M., Broaddus, C., and Myers, G. (2018). Cell detection with star-convex polygons. arXiv [cs.CV]. https://doi.org/10.1007/978-3-030-00934-2_30.

2. Bankhead, P., Loughrey, M.B., Fernández, J.A., Dombrowski, Y., McArt, D.G., Dunne, P.D., McQuaid, S., Gray, R.T., Murray, L.J., Coleman, H.G., et al. (2017). QuPath: Open source software for digital pathology image analysis. Sci. Rep. *7*, 16878.

3. Stringer, C., Wang, T., Michaelos, M., and Pachitariu, M. (2021). Cellpose: a generalist algorithm for cellular segmentation. Nat. Methods *18*, 100–106.

4. Hickey, J.W., Tan, Y., Nolan, G.P., and Goltsev, Y. (2021). Strategies for accurate cell type identification in CODEX multiplexed imaging data. Front. Immunol. *12*, 727626.

5. Zhang, W., Li, I., Reticker-Flynn, N.E., Good, Z., Chang, S., Samusik, N., Saumyaa, S., Li, Y., Zhou, X., Liang, R., et al. (2022). Identification of cell types in multiplexed in situ images by combining protein expression and spatial information using CELESTA. Nat. Methods *19*, 759–769.

6. Shaban, M., Bai, Y., Qiu, H., Mao, S., Yeung, J., Yeo, Y.Y., Shanmugam, V., Chen, H., Zhu, B., Weirather, J.L., et al. (2024). MAPS: pathologist-level cell type annotation from tissue images through machine learning. Nat. Commun. *15*, 28.
